# Enhancing bile tolerance improves survival and persistence of *Bifidobacterium *and *Lactococcus *in the murine gastrointestinal tract

**DOI:** 10.1186/1471-2180-8-176

**Published:** 2008-10-09

**Authors:** Debbie Watson, Roy D Sleator, Colin Hill, Cormac GM Gahan

**Affiliations:** 1 Alimentary Pharmabiotic Centre, University College Cork, Cork, Ireland; 2 Department of Microbiology, University College Cork, Cork, Ireland; 3 School of Pharmacy, University College Cork, Cork, Ireland

## Abstract

**Background:**

The majority of commensal gastrointestinal bacteria used as probiotics are highly adapted to the specialised environment of the large bowel. However, unlike pathogenic bacteria; they are often inadequately equipped to endure the physicochemical stresses of gastrointestinal (GI) delivery in the host. Herein we outline a patho-biotechnology strategy to improve gastric delivery and host adaptation of a probiotic strain *Bifidobacterium breve *UCC2003 and the generally regarded as safe (GRAS) organism *Lactococcus lactis *NZ9000.

**Results:**

*In vitro *bile tolerance of both strains was significantly enhanced (*P *< 0.001), following heterologous expression of the *Listeria monocytogenes *bile resistance mechanism BilE. Strains harbouring *bilE *were also recovered at significantly higher levels (*P *< 0.001), than control strains from the faeces and intestines of mice (*n *= 5), following oral inoculation. Furthermore, a *B. breve *strain expressing *bilE* demonstrated increased efficacy relative to the wild-type strain in reducing oral *L. monocytogenes *infection in mice.

**Conclusion:**

Collectively the data indicates that bile tolerance can be enhanced in *Bifidobacterium *and *Lactococcus *species through rational genetic manipulation and that this can significantly improve delivery to and colonisation of the GI tract.

## Background

Probiotics, defined by a working group of the International Life Sciences Institute Europe (ILSI Europe) as "a viable microbial food supplement, which beneficially influences the health of the host" [1], have become the focus of considerable research interest in recent years [2-4]. Live probiotic organisms (including *Bifidobacterium *spp.) have been shown to reduce the symptoms of inflammatory conditions such as inflammatory bowel disease (IBD) and irritable bowel syndrome (IBS) [5]. This is believed to occur *via *the localized stimulation of anti-inflammatory cytokines (including IL-10) as a result of beneficial alterations to the microbiota [6]. Proof of principle and efficacy has been demonstrated with the inert food organism *Lactococcus lactis *which has been engineered to secrete IL-10 locally within the gut in murine models of IBD in order to alleviate symptoms of gastrointestinal inflammation [7]. However, despite their obvious clinical potential, these strains are often poorly adapted to conditions encountered in the upper gastrointestinal tract and delivery of viable organisms from the mouth to the large intestine remains a major hurdle to their use in human therapies [8,9].

*Listeria monocytogenes *is a Gram-positive intracellular foodborne pathogen capable of withstanding a variety of hostile environmental conditions, including the numerous stresses encountered during the production, preparation, and storage of food [10]. Following consumption *L. monocytogenes *effectively survives the extreme conditions encountered during gastric passage, including the low pH of the stomach, low oxygen content and elevated osmolarity and bile salts associated with the upper small intestine [10,11]. Bile in particular represents a key challenge to bacteria that survive and transit the stomach and enter the small intestine. *Listeria *has been isolated from the human gallbladder [12,13], indicating an inherent ability to tolerate high concentrations of bile. Previously Sleator *et al*., [14], identified a novel bile resistance mechanism designated BilE, which when disrupted resulted in a bile sensitive phenotype. BilE, which functions by excluding bile from the cell, has also been shown to facilitate improved gastrointestinal transit in mouse models of infection and as such contributes to the gastrointestinal phase of *L. monocytogenes *infection. Interestingly, not many homologues of the *bilE *operon have been identified in any of the genomes of the probiotic or commensal organisms sequenced to date. The only homology found was *busA *(*opuA*) of *L. lactis*, which encodes a glycine betaine uptake system. This system is osmotically inducible as was believed to be the case with *bilE*. However, Sleator et al, (2005) have shown that *bilE* does not play a role in osmotolerance, but in fact a major role in bile tolerance.

Herein, we demonstrate that heterologous expression of the listerial bile resistance mechanism BilE improves bile resistance *in vitro *as well as enhancing gastrointestinal persistence and clinical efficacy of the probiotic strain *B. breve *UCC2003. Furthermore, expression in *L. lactis *enhanced bile tolerance and *in vivo *survival and may have applications in targeted vaccine or drug delivery by this organism.

## Methods

### Bacterial strains, plasmids, and culture conditions

The strains and plasmids used in this study are listed in Table 1. *Escherichia coli *strain DH10B (Invitrogen, Paisley, United Kingdom) used as a cloning host, was grown aerobically at 37°C in Luria-Bertani (LB) medium [15].*Bifidobacterium breve *UCC2003, a human commensal originally isolated from an infant nursling stool, was anaerobically cultured in Reinforced Clostridial medium (Oxoid) or de Man, Rogosa and Sharpe medium (MRS) supplemented with 0.05% (wt/vol) l-cysteine HCl. Anaerobic conditions were maintained using an Anaerocult oxygen-depleting system (Merck, Darmstadt, Germany) in an anaerobic chamber. *Lactococcus lactis *NZ9000 strain was grown in M17 medium supplemented with 0.5% (wt/vol) glucose (GM17) at 30°C. *L. monocytogenes *EGD-e [16] was cultured aerobically at 37°C in brain heart infusion (BHI) medium. Oxoid Ltd., (Basingstoke, Hampshire, United Kingdom) supplied all growth media. For solid media, 1.5% agar was added. When appropriate, antibiotics were added at the following final concentrations: for *E. coli*, *B. breve*, and *L. lactis*, chloramphenicol 15 μg ml^-1^, 4 μg ml^-1 ^and 5 μg ml^-1 ^respectively. Porcine bile (B-8631) was obtained from Sigma (Sigma Chemical Co. Ltd., Poole, United Kingdom). The required quantity of bile was solubilised in water and autoclaved.

**Table 1:** Bacterial strains, plasmids and primers used in this study

**Strain or plasmid**	Relevant properties*	**Source or Reference**
**Strains**		
		
***B. breve***		
UCC2003	Wild-type parent strain	UCC culture collection
UCC2003-*bilE*^-^	UCC2003 containing the cloning vector pNZ8048	This study
UCC2003-*bilE*^+^	UCC2003 containing the cloning vector pNZ8048-*bilE*^+^	This study
		
***L. lactis *subsp. *Cremoris***		
NZ9000	*L. lactis *subsp. *cremoris *MG1363 carrying nisRK on the chromosome	10.
NZ9000-*bilE*^-^	NZ9000 containing the cloning vector pNZ8048	This study
NZ9000-*bilE*^+^	NZ9000 containing the cloning vector pNZ8048-*bilE*^+^	This study
		
***E. coli***		
DH10B	Cloning host	Invitrogen
		
***L. monocytogenes***		
EGD-e	Wild-type of serotype 1/2a for which the genome sequence is available.	UCC culture collection
		
**Plasmid**		
pNZ8048	*Cm^r^, low copy number plasmid.	37.
pNZ8048-*bile*^+^	*Cm^r^, low copy number plasmid, harbouring *bilE *under the control of the native listerial promoter.	This study
		
**Primers**	**Sequence 5'-3'**	
BilEF	CATTCTAGAGTTTGTAAGTTATT	
BilER	CAAATTCTTTGTTGAATTCCTGCAGATAT	
Bif16SF	CCGGATGCTCCATCACAC	
Bif16SR	ACAAAGTGCCTTGCTCCCT	
Lac16SF	TGGCTCAGGACGAACGCTGGCGGC	
Lac16SF	CCTACTGCTGCCTCCCGTAGGAGT	
RT-PCRF	GCTTGATTCCACTGACAACTGG	
RT-PCRR	CTTGTGGTGTTGCTACTTGGAC	

### Genetic manipulations

Plasmid DNA was isolated from *E. coli *using a QIAprep Spin Miniprep kit according to the manufacturer's instructions (QIAGEN, Crawley, United Kingdom). Genomic DNA was isolated from *L. monocytogenes *EGD-e using a Genelute bacterial genomic DNA kit (Sigma, Steinheim, Germany) as recommended by the manufacturer. Transformation of *L. lactis *was achieved according to the protocol of de Vos *et al*. [17]. Standard procedures were used for DNA manipulation in *E. coli *[15]. Restriction endonucleases (Roche Diagnostics, Mannheim, Germany), T4 DNA ligase (Roche), and 2× PCR mixture (Promega, Madison, WI) were used as recommended by the manufacturers. Oligonucleotide primers were synthesized by Sigma Genosys (Haverhill, United Kingdom). PCR products required for cloning were generated with KOD hot-start high-fidelity DNA polymerase (Merck, Nottingham, United Kingdom) using 10 ng *L. monocytogenes *genomic DNA.

### Plasmid construction

Analysis of the *bilE *operon in *L. monocytogenes *EGD-e (accession number NC_003210) uncovered the presence of two Open Reading Frames (ORFs) *bilEA *(accession number NP_464946 and GI number GI:16803461) and *bilEB *(accession number is NP_464947.1 and GI number is GI:16803462), oriented in the same direction and overlapping by five nucleotides. PCR primers with incorporated *Xba*I (5'-CATTCTAGAGTTTGTAAGTTATT-3') and *Pst*I (5'-CAAATTCTTTGTTGAATTCCTGCAGATAT-3') restriction enzyme sites (underlined) were used to amplify the complete *bilE *operon from the chromosome of *L. monocytogenes *EGD-e. The resultant 2.9 kb PCR product was digested with *Xba*I and *Pst*I and subsequently ligated into similarly digested pNZ8048 using T4 DNA ligase (Roche Diagnostics). The resultant plasmid, containing *bilE *under the transcriptional control of its own promoter, was designated pNZ8048-*bilE*^+ ^as in Table 1. Both pNZ8048-*bilE*^+ ^and pNZ8048-*bilE*^- ^(control) initially introduced into *E. coli *DH10B as a cloning host. Plasmid DNA was extracted from successful transformants, was sequenced and subsequently transformed into the bifidobacterial strain UCC2003 and lactococcal strain NZ9000 yielding UCC2003-*bilE*^+^and NZ9000-*bilE*^+ ^respectively. Strains harbouring pNZ8048, which was used as a negative control, were named UCC2003-*bilE*^- ^and NZ9000-*bilE*^- ^respectively. Chloramphenicol was added to plates as a selective marker.

### Electroporation

Electroporation of plasmid DNA into *E. coli *and *L. lactis *was performed essentially as previously described. Electro-transformation of *B. breve *with plasmid DNA was performed as described by MacConaill *et al. *[18]. Essentially, mid-logarithmic-phase cells (optical density at 600 nm, 0.5 to 0.6) were chilled on ice for 20 min, followed by centrifugation. The cell pellet was washed twice and resuspended in 0.5 M sucrose-1 mM citrate buffer (pH 5.8). Cells were incubated on ice for 10 min, and this was followed by electro-transformation with a Bio-Rad Gene Pulser II apparatus under the following conditions: 25 μF, 200 Ω and 2.0 kV cm^-1^. Modified Rogosa medium was added to the cells, and the mixture was incubated anaerobically at 37°C for 2.5 hr prior to plating onto RCA (Reinforced Clostridial agar) containing the appropriate antibiotic.

### Plasmid stability study

*B. breve *UCC2003 colonies containing pNZ8048-*bilE*^+ ^and pNZ8048-*bilE*^- ^and *L. lactis *NZ9000 colonies containing pNZ8048-*bilE*^+^and pNZ8048-*bilE*^- ^were first cultured in MRS broth containing 4 μg ml^-1 ^chloramphenicol and GM17 broth with 5 μg ml^-1 ^respectively. Cells were then sub-cultured in fresh MRS and GM17 broth without antibiotic selection every 24 hrs for a total of 50 generations. Vector segregation stability was monitored by plating isolated colonies every 48 hrs. 100 colonies were replica plated onto MRSCm4 and GM17Cm5. *B. breve *was incubated anaerobically at 37°C and *L. lactis *incubated aerobically at 30°C for 24 hrs. The percentage of loss of the test plasmid in the population was then calculated.

### Transcriptional analysis

Total RNA was isolated from *B. breve *UCC2003 and *L. lactis *NZ9000 cells grown to an optical density at 600 nm (OD_600_) of 0.6 using the macaloid acid method as described by Ventura *et al*. (38) and then treated with DNase (Roche). Reverse transcription for cDNA was mediated by thermostable Superscript reverse transcriptase (Invitrogen) using 10–20 μg RNA as a template in a 30 μl reaction mixture containing 6 μl 5× Superscript III buffer, 2 ng of random primers p(dN)_6_, 3 μl 100 mM dithiothreitol, 1.2 μl of deoxynucleoside triphosphate mix and the Superscript enzyme (Invitrogen, Paisley, United Kingdom), which was used according the manufacturer's instructions to produce cDNA. The cDNA generated was used as a template for reverse transcription (RT)-PCRs performed with primers RT-PCRF and RT-PCRR. In all cases, control PCR reactions were used to ensure the complete removal of DNA from RNA preparations prior to reverse transcription.

### Resistance to Bile

To determine the ability of strains to survive at sub-lethal bile concentrations, the *B*. *breve *and *L. lactis *cultures were grown to stationary phase and were subsequently inoculated (3%) into MRS and GM17 broth containing 1% (wt/vol) porcine bile. Viable cell counts were performed at intervals by serial dilution in one-quarter-strength Ringer's solution and plating onto RCMCm4 or GM17Cm5 respectively.

### Animal studies

Female BALB/c (Harlan UK Ltd. Bicester, Oxon, United Kingdom) mice (aged 8–12 weeks) were used for *in vivo *studies. Mice were housed in pathogen free conditions in a dedicated facility and were fed standard laboratory feed and water *ad libtium*. All animal procedures were performed according to the University's ethical guidelines. Inoculations were carried out essentially as described by Sleator *et al*., [19].

### Probiotic Gastrointestinal Persistence studies

**Figure 1:**
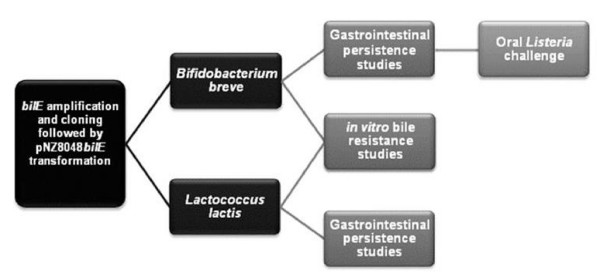
Schematic of the design protocol.

Prior to commencement of the trials (outlined in Fig. 1), the absence of background microflora in murine faecal pellets was confirmed by plating onto the two selective agars containing antibiotic. For the study five mice were used per bacterial strain. Each mouse was administered 20 μL of *bifidobacteria *or *lactococcus *(2 × 10^9 ^CFU per mouse) by oral pipette for 3 consecutive days, leading to colonization of the gastrointestinal tract. Gastrointestinal persistence of both strains was tracked by measuring the excretion of viable *B. breve *in fresh faecal samples collected for 19 days post feeding and *L. lactis *for 3 days. Fresh samples were weighed, homogenized in PBS (Gibco, Paisley, Scotland), diluted and plated onto RCA or GM17 with the appropriate level of chloramphenicol. At days nineteen (*B. breve*) and four (*L. lactis*) post dosing the mice were sacrificed by cervical dislocation, intestines and caeca were excised. The organs were homoginized in 5 mls PBS, serially diluted and the bifidobacterial and lactococcal numbers in the intestines and caeca of dosed animals were determined by spread-plating homogenates onto RCACm4 and GM17Cm5 plates.

### *Listeria *challenge

Animal feeding trials were performed to determine whether the probiotic strains harbouring pNZ8048-*bilE*^+ ^conferred protection against subsequent infection with *L. monocytogenes*. BALB/c mice were fed Bifidobacteria (2 × 10^9 ^CFU per mouse) by oral pipette for 3 consecutive days before oral infection with *L. monocytogenes *EGD-e::pPL2*lux *(2 × 10^10 ^CFU per mouse) on day 14. Three days post infection, the mice were sacrificed by cervical dislocation, livers and spleens were excised, homogenized in 5 mls PBS, and serial dilutions were plated onto BHICm agar, which was followed by overnight incubation at 37°C. The resulting colonies were used to calculate the number of bacteria per organ.

### Statistical Analysis

Numerical results were expressed as arithmetic means +/- standard deviations of the means. CFU determinations were converted to Log_10 _values, and then the arithmetic means and standard deviations were calculated. Error bars in the figures represent standard deviations. Student's *t *test was performed to determine the statistical significance.

## Results

### Improving tolerance to porcine bile

**Figure 2:**
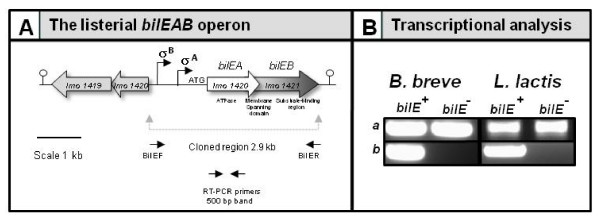
**(A) Flow diagram for the expression of the *bilEAB *operon, indicating the listerial *bilE *operon** (B) RT-PCR analysis of the heterologous expression of the *bilEAB *bile resistance loci in *B. breve *and *L. lactis *(a) Control PCRS using cDNA template and the 16S RNA specific primers Bif16SF, Bif16SR, Lac16SF and Lac16SR to confirm the presence of the correct template. (b) *bilE *specific primers RTPCR F and RTPCR R amplify a 500 bp fragment from both UCC3003-*bilE*^+ ^and NZ9000-*bilE*^+ ^cDNA, while as expected no product was obtained when the cDNA of the control strains UCC3003-*bilE*^- ^and NZ9000-*bilE*^- ^were used as template.

**Figure 3:**
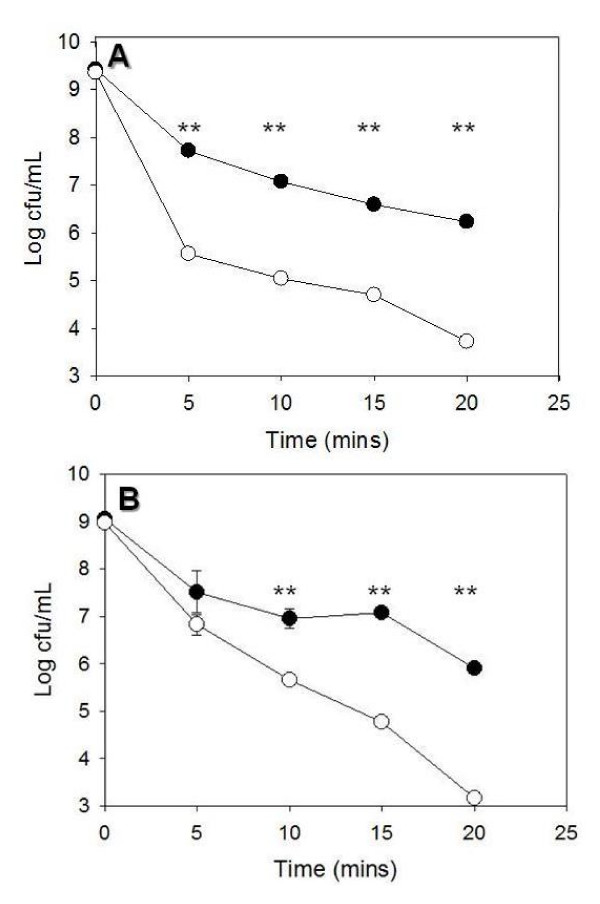
**Survival of stationary phase *Lactococcus lactis *(A) and *Bifidobacterium breve *(B) in 1% porcine bile.** pNZ8048-b*ilE*^+ ^(black circles) and pNZ8048-*bilE*^- ^(white circles). Overnight cultures were inoculated (3%) into GM17 and MRS broth containing 1% porcine bile. Viable cell counts were performed by serial dilution in one-quarter strength Ringer's solution followed by plating onto GM17Cm5 or RCMCm4 respectively. **; P < 0.001 compared with the control. Standard deviations of triplicate results are represented by error bars.

The BilE system was amplified from *L. monocytogenes *and cloned into the plasmid vector pNZ8048 under the control of its native promoter. The plasmid was electroporated into both *B. breve *UCC2003 and *L. lactis *NZ9000 (see Table 1). To establish the segregational stability of the pNZ8048 constructs a plasmid stability assay was performed over approximately 50 generations. Both constructs (pNZ8048-*bilE*^+ ^and pNZ8048-*bilE*^-^) were capable of replication on *B. breve *UCC2003 and were found to exhibit considerable stability (98.55 +/- 0.63%) in the absence of selective pressure (data not shown). The constructs were also capable of replication in *L. lactis *NZ9000 and were found to exhibit considerable stability (100%) in the absence of selective pressure. Proof that the operon was heterologously expressed in both bacterial strains was evident as a single mRNA transcript was obtained by reverse transcription polymerase chain reaction (RT-PCR) analysis (Fig. 2) also *in vitro *studies showing increased bile resistance supports this by indicating that the bile resistance locus was functional. Due to difficulty in obtaining human bile; porcine bile was used to examine the bile tolerance of *B. breve bilE*^+ ^and *L. lactis bilE*^+^. Porcine bile is considered an acceptable substitute because the salt/cholesterol, phospholipids/cholesterol and glycine/taurine ratios resemble the composition of human bile [20]. We utilised levels of porcine bile (1% w/v) that were lethal for the bacterial species examined and are likely to approximate *in vivo *levels in regions of the small intestine (e.g. the duodenum) where bile is most concentrated [20]. It was observed that the presence of *bilE *rendered the engineered *L. lactis *strain considerably more resistant to the initial kill by porcine bile relative to the wild-type (empty pNZ8048). The engineered strain demonstrated a 2.5-log enhanced resistance to bile over the 20 minute course of the kill curve (Fig. 3A). In contrast whilst *B. breve bilE*^+ ^demonstrated a similar initial kill to the wild-type (after 5 minutes) the engineered strain was subsequently better equipped to survive in porcine bile. At 15 minutes and 20 minutes post-exposure the *B. breve bilE*^+ ^strain demonstrated 2.5 log enhanced survival (Fig. 3B). Similar results were seen in experiments carried out using bovine bile (oxgall) (data not shown).

### Gastrointestinal Persistence of *L. lactis *BilE^+ ^and *B. breve *BilE^+^

In order to test whether the elevated bile tolerance of the engineered strains would influence survival in the GI tract, we utilized a mouse model to follow survival *in vivo*, using an approach previously outlined by Sheehan *et al*. [21]. To facilitate gastrointestinal colonization by *B. breve *and *L. lactis *strains, each BALB/c mouse was orally administered 2 × 10^9 ^CFU for three successive days. Gut persistence was monitored by faecal carriage with samples taken every 24 h for 3 days in the case of *L. lactis *and every 48 h for 19 days for *B. breve*.

**Figure 4:**
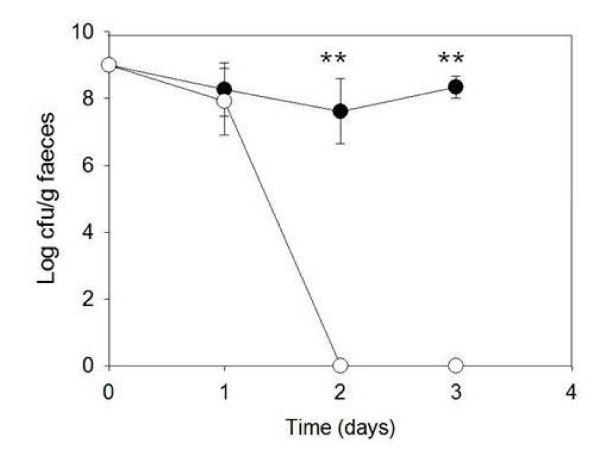
**Effect of *bilE *on gastrointestinal persistence over 3 days.***Lactococcus lactis bilE*^+ ^(black circles) and *Lactococcus lactis bilE*^- ^(white circles) were used for peroral inoculation of female BALB/c mice (n = 5). *Lactococcus lactis *counts were determined in stools at 24, 48 and 72 hour intervals. Error bars represent the standard deviation of five animals. **; P < 0.001 compared with the control.

*L. lactis *BilE^+ ^demonstrated significantly enhanced *in vivo *survival relative to the control over the course of the study (Fig. 4). Both BilE^+ ^and BilE^- ^strains were detectable in faeces at approximately the same levels at 24 hours post inoculation. However, the wild-type was undetectable at day 2 post-inoculation. This reflects the naturally poor rate of gastrointestinal survival for *L. lactis *also observed in other studies [22]. In contrast *L. lactis *BilE^+^was detectable at high levels in murine faeces at days 2 and 3 post-inoculation. Neither BilE^+ ^nor BilE^- ^strains engineered *L. lactis *were detectable at day 4 post-inoculation. These data demonstrate significant (P < 0.001) enhancement of gastrointestinal survival of *L. lactis *through rational biological engineering.

**Figure 5:**
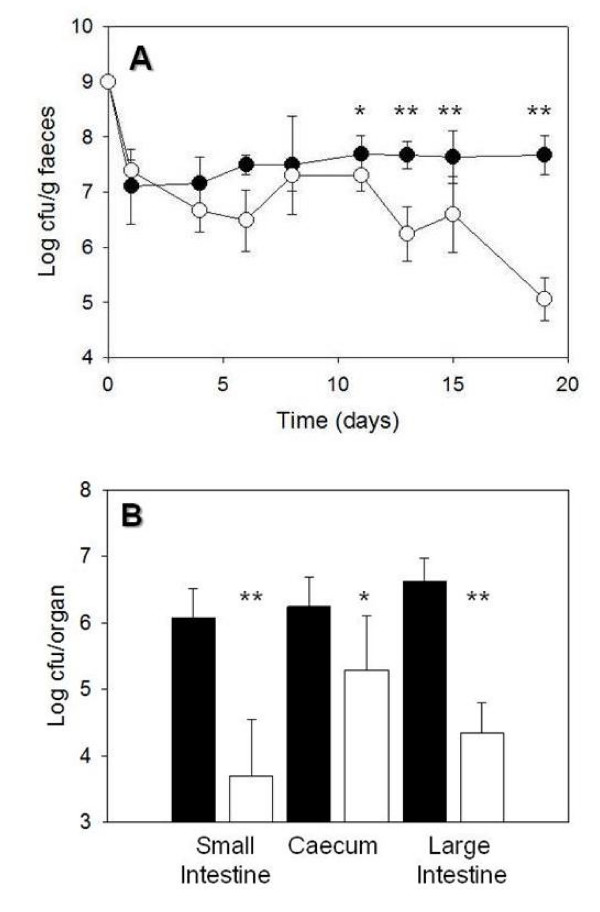
**(A) Effect of *bilE *on the gastrointestinal persistence of *Bifidobacterium breve bilE*^+ ^(black circles) and *Bifidobacterium breve bilE*^- ^(white circles) were used for peroral inoculation of female BALB/c mice (n = 5).***Bifidobacterium breve *counts were determined in stools at 48 hour intervals. (B) At day 19 mice were sacrificed and *Bifidobacterium breve *harbouring *bilE *(black bars) were recovered at significantly higher numbers in the intestines and caeca than the controls (white). *; *P *< 0.01, **, *P *< 0.001.

*B. breve *UCC2003 transiently colonized the murine GI tract at levels of approximately 10^7 ^CFU/g faeces irrespective of the presence of BilE. However, at day 12 post inoculation a significant (P < 0.001) difference began to emerge between the engineered *B. breve *UCC2003 BilE^+ ^strain and the BilE^-^, with the BilE^+ ^strain demonstrating elevated persistence in faeces compared to the wildtype, which declined significantly (Fig. 5A). At the final day of the experiment (day 19 post inoculation) the BilE^+ ^strain persisted at levels of approximately 4.5 × 10^7 ^CFU/g faeces whereas the wild-type had declined to 1 × 10^5 ^CFU/g faeces. This is a more substantial difference than that observed by Sheehan *et al. *[21] using a similar approach which involved cloning the BetL osmotolerance locus into *B. breve*.

At day 19 mice were examined directly for the presence of *B. breve *UCC2003 in the small intestine, caecum and large intestine (Fig. 5B). We confirmed a 2-log difference between the BilE^+ ^and *wt *strains in the small intestine with the engineered strain present at significantly higher levels. This finding is important given that this region is associated with the highest levels of bile in the GI tract [20]. Interestingly Sheehan *et al*. [21] did not demonstrate enhanced survival of the BetL^+ ^strain in the small intestine. We also determined significantly enhanced levels of the engineered strain in the caecum and large intestine (P < 0.001). Overall, the approximately 2 log increased persistence of the engineered strain in the murine intestine mirrored the results of the faecal persistence studies.

### Effect of engineered BilE^+ ^*B. breve *on oral *Listeria *challenge

**Figure 6:**
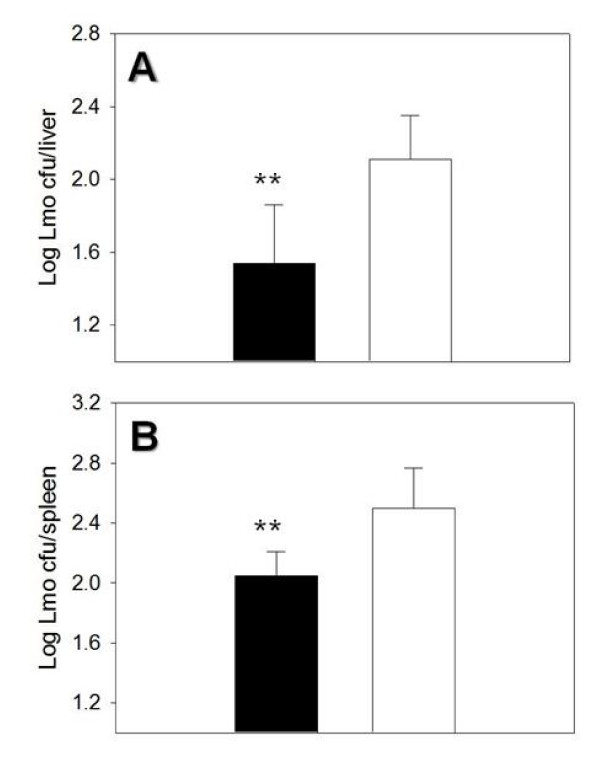
**Improved clinical efficacy.** Probiotic dosing of BALB/c mice with *Bifidobacterium breve bilE*^+ ^(black) reduces the level of subsequent *Listeria monocytogenes *infection. Bacterial growth was followed in (A) the liver and (B) the spleen 3 days post infection.

Administration of *B. breve *UCC2003 has previously been demonstrated to reduce splenic levels of *L. monocytogenes *following oral infection of mice [23]. We hypothesized that the elevated colonisation of *B. breve *BilE^+ ^in the small intestines of mice (Fig. 5) may further enhance this protective effect since *L. monocytogenes *invades at this site. To test this hypothesis we administered mice with either wild-type or BilE^+ ^*B. breve *UCC2003 on three consecutive days and challenged with oral *L. monocytogenes *infection at day 14 post-inoculation. At the 14 day time point we again detected higher levels of the engineered BilE^+ ^strain relative to the wild-type in mouse faeces (data not shown). Following oral infection with *L. monocytogenes *we determined that oral dosing with the engineered BilE^+ ^*B. breve *strain significantly enhanced clearance of *L. monocytogenes *as assessed by pathogen load in the liver (Fig. 6A). A similar (though not statistically significant) trend was seen when the numbers of *Listeria *in the spleens of the infected mice was determined (Fig. 6B). Although differences in infectious burden were small the data indicate improved probiotic efficacy of engineered *B. breve*.

## Discussion and conclusion

Foodborne pathogens are capable of rapid adaptation to diverse environmental niches associated with survival in the external environment and transit through the gastrointestinal tract of the host [24]. In contrast, the autochthonous gastrointestinal microbiota demonstrates a high degree of niche-specialisation [25]. This specialisation is an impediment to the delivery of so-called probiotic strains to the human GI tract as these strains must survive gastric acid in the stomach and elevated osmolarity and bile acids in the small intestine before establishing a presence in the large bowel [26]. The term 'patho-biotechnology' was recently coined to highlight the beneficial biotechnological uses of molecular systems derived from pathogens [24,27-29]. This concept was recently applied to improve the osmotolerance of potential probiotic strains through rational molecular engineering [21,30]. Here we implement the patho-biotechnology concept to improve the *in vivo *and *in vitro *bile resistance of a potential probiotic strain, *B. breve *UCC2003, as well as the food isolate *L. lactis*, through cloning and expression of a bile tolerance system in these organisms.

Bile acids are amphipathic molecules synthesized from cholesterol that are produced in the liver, stored interdigestively in the gall bladder and released into the duodenum where their main function is the breakdown of dietary fats. Bile also plays a vital role in the physiochemical defences of the host through direct degradation of bacterial membranes [30]. Selection of probiotic strains for use in humans has typically been based upon intrinsic acid and bile resistance traits [31]. However, members of the *Lactobacillus *and *Bifidobacteria *spp are known to demonstrate low levels of innate bile tolerance when compared to pathogens such as *L. monocytogenes *[20]. We recently described the presence of a novel bile exclusion system (BilE) in *L. monocytogenes *which permits the active exclusion of bile from the cell in a manner similar to multi drug efflux pumps of Gram negative bacteria such as *E. coli*. In *Listeria *elimination of BilE results in a 5-log reduction in tolerance of bile *in vitro *and has a significant impact upon virulence potential [14]. BilE is not widely distributed in Gram positive organisms; however homology was found with the osmotically inducible *busA *of *L. lactis*. Sleator *et al.*, 2005, showed that bilE is involved in bile exclusion and not in osmotolerance as previously believed. Here cloning and expression of BilE in *B. breve *and *L. lactis *resulted in a 2.5 to 3.5-log increased tolerance of porcine bile. Similarly these engineered strains demonstrate improved resistance to bovine bile *in vitro *(data not shown). These data provide proof of concept that bile tolerance in these strains can be significantly enhanced through rational genetic manipulation.

Wild-type *L. lactis *survives poorly in the murine GI tract [22]. However, *L. lactis *has been proposed as a vehicle for delivery of oral vaccines and biotherapeutic agents [9]. Indeed, an *L. lactis *strain engineered to produce human IL-10 has recently been investigated in human clinical trials for the therapy of Crohn's disease [32]. We show that *L. lactis *expressing cloned BilE demonstrates significantly enhanced survival in the GI tract of mice (as measured in faeces) relative to the wild-type *L. lactis *strain. The results indicate that molecular engineering approaches may have the potential to enhance the proposed biomedical applications of *L. lactis*.

Survival of *B. breve *in the murine GI tract was also significantly enhanced through expression of BilE. The engineered strain was detected at significantly higher levels than the wild-type in faeces (at day 12) as well as in the small intestine, caecum and colon of inoculated animals. *Bifidobacterium *species are known to only transiently colonise the GI tract when administered as a probiotic therapeutic in experimental animals [33] and humans [34]. Here we show that increased bile tolerance can significantly impact on survival and colonisation. It is likely that enhanced persistence will also enhance efficacy in therapeutic models. Indeed, in support of this we demonstrate that bifidobacteria with increased bile tolerance are capable of reducing oral infection with *L. monocytogenes*. Whilst the levels of enhanced protection are low (<1 log reduction in *Listerial *invasion of internal organs relative to mice administered the wild-type) they are statistically significant.

We recently demonstrated that cloning and expression of the betaine uptake system BetL in *B. breve *UCC2003 can significantly enhance acid and salt tolerance of the engineered strain and promote survival in the mouse GI tract [21]. We determined moderately higher rates of survival *in vivo *when strains are engineered to enhance bile tolerance. Indeed, we find greater colonisation of the small intestine through enhanced bile tolerance of *B. breve*, a finding that may have significant biomedical consequences since the small intestine is the key site of immune sampling in the GI tract. In addition, we have extended the earlier study to demonstrate an application in the GRAS organism *L. lactis*. This is particularly significant given that *L. lactis *heterologously expressing the cytokine IL-10 locally within the gut in murine models of IBD have previously been shown to alleviate symptoms of gastrointestinal inflammation [8]. Thus, any attempt to increase the GIT persistence of this strain may be important for the future development of novel vaccine and drug delivery systems. Collectively this work outlines the application of the patho-biotechnology concept to enhance the robustness and efficacy of potential probiotic organisms. Whilst this approach is unlikely to gain immediate acceptance by regulatory bodies given the genetically modified nature of the constructed strains, we propose that this work represents a further proof-of-concept that may inform future studies to enhance delivery of live organisms, vaccines or biotherapeutic agents to the GI tract [35,36].

## Authors' contributions

DW, RDS, CGMG and CH participated in the design and coordination of the study and the drafting of the manuscript. DW and RDS carried out the main experimental work. All authors have read and approved the final manuscript.
